# The Preservation of the Eyes

**Published:** 1853-03

**Authors:** 


"You should never touch your eye but with your elbow."—Old Proverb.

*The Preservation of the Eyes.*—There is a tradition at least as old as the Talmud, that the eyes are strengthened by drawing the fingers across the eyelids in a horizontal direction. Ex-President Adams, who was affected with an obstruction of the tear-passage, used this method to get rid of the accumulated fluid, and the ancient practice was brought into greater notice by the example of the illustrious statesman. The obsolete theory, that the anterior surface of the eye-ball becomes flattened as age advances, was again revived, and it became a business to advertise instructions for kneading the organ into shape with the fingers! For the very moderate sum of ten dollars, the tell-tale spectacles might be laid aside, and ancient ladies and gentlemen be enabled to sew and read with all the sharpness of a miss in her teens. If we may credit the advertisements in newspapers, and who dare disbelieve them, the ten-dollar professor met with marvellous success; yet an improvement was made in the manner of increasing the rotundity of the eye by placing over it a wooden cup attached to an india rubber bottle, similar to the ordinary breast pump! On the appearance of the new method of altering the shape of the eye, the fee was diminished, when the original candidate for public favor offered his services for half price. In the hands of a good juggler, experiments with cups and balls seem almost miraculous, and it is not surprising that new arrangements may produce new wonders. The machine described by Captain Marryatt, for altering the disposition of an individual by placing exhausters on the protuberances of the skull, has not yet been turned to practical advantage. Some self-styled professor will, it is presumed, shortly take the matter in hand, and advertise instructions by which an easy change of temper may be effected.

It cannot be expected that operations founded on a false theory can be safe in practice. It is untrue that the outer surface of the eye becomes flatter with advancing age, and therefore manipulations to restore what is not wanting, in an organ so delicate in structure that a rude push may be followed by perpetual darkuess, should be avoided. The principal lens of the eye is situated behind the pupil, and kept in proper position by membranes finer than the finest gold-beater's skin. These delicate membranes are liable to be ruptured by blows, falls, or other causes, and the lens, which is naturally clear as crystal, becomes white and opaque. Opacity of the lens, or what is called cataract, may be produced without laceration of the membranes, by merely interfering with the circulation of the vessels which supply it. The writer was lately called to visit an aged female who had been suffering acutely for months, after submitting, while in health, to the manipulations of a rejuvenating itinerant. The lens was dislocated and pressed on the sensitive nerve at the margin of the pupil. The pain occasioned by pressure of this kind may be compared to that produced by pressing the exposed nerve of a tooth with a tooth-pick, but in the former case the pain is continuous and not so easily removed as in the latter. Other cases of injury attributed to manipulation, such as cross eyes, double vision, &c., have come under the writer's notice. Last month, in presence of the editor, he operated for cataract in the case of a lady, whose vision with the aid of spectacles was perfect until she was induced by plausible advertisements to pay for a course of lessons. After the third lesson, vision became indistinct, and blindness ultimately followed. Beer was called to examine a gentleman who had always enjoyed excellent sight until it was lost in a moment: The patient had been at a party of friends, when a person stepped suddenly behind him, and covering both eyes with his hands, wished him to guess who it was. The former, without speaking a word, endeavored to escape from the pressure, and when the eyelids were opened he was entirely bereft of sight.

Although there was not the least appearance of injury, the sufferer remained hopelessly blind. From this melancholy example, Beer concludes that the eyes are liable to injury even from moderate pressure.

This delicate organization may be deranged even by medicines. Dr. Currier relates that owing to the spasm produced by strychnia and veratria, the capsule has been lacerated, and the lens dislocated. Others have noticed the occurrence of cataract from the operation of medicines. It has been known to follow what is called the aconitine trick.

The chronometer of a mariner is inclosed in a case, and this is again packed in a box, by which it may be further protected from concussion. Should he who depends on this instrument for ascertaining the longitude, unnecessarily interfere with the delicate springs regulating its movements, he will, it is probable, be mistaken in his reckoning, or more probably still, the works will be so deranged that he will be deprived of its services altogether. Although capable, with fair play, of remarkable endurance, the eye is finer than the finest chronometer. It is protected above by the eyebrow; below by the cheek; on one side by the nose, and on the other by the temple. The hairs of the eyebrow are so arranged that water falling on the upper portion of the head is directed away from the eye in the most careful manner. When there are such extraordinary pains for its preservation, there should be some hesitation before unnecessary or injudicious interference with an organ of such inestimable value.

There is a popular notion, sanctioned even by medical men who ought to know better, that the eyes are preserved by opening them every morning in a basin of cold water. Some of the worst cases of pterygium, or film on the surface of the eye, have been witnessed in those who boasted of this practice. When a drop of water gets into the windpipe, the nostril, or the ear, irritation is produced, and when the eyes are opened under water the sensation is anything but agreeable. The eye is lubricated by a secretion admirably adapted to facilitate the motion of the lid over its surface; and as this secretion is partially soluble in water, it is as inconsistent with common sense to wash it away as it is to remove the oil from the wheels of machinery. It is unquestionably important that the cleanliness of the organ be maintained; yet this may be accomplished in the usual manner, without opening the lubricating surfaces. When the secretion is vitiated by cold or other causes, quince-seed tea or milk and water are preferable for ablution to water alone. Avoid eye-waters, many of which contain lead, or there are ten chances to one they will produce an incurable film. To make this clear, dissolve a little sugar of lead in water and pour the transparent solution in a wine glass containing a watery solution of common salt. When the fluids are mixed, a white precipitate of chloride of lead falls to the bottom of the glass. When the eye-waters containing lead are permitted to pass to the surface of the eye, the tears furnish common salt, and the lead is precipitated. The transparent portion of the eyes is sometimes extensively tattooed with this white leaden powder, and vision becomes indistinct or even destroyed.

When the general health is robust it is astonishing what an amount of labor the organs of vision will endure; yet when it is depressed, especially by mental disturbance during a periodical function, they are easily deranged by too close application to business. When they have become weak, much for their preservation depends on the proper management of the light to which they are exposed. When the light is in excess, it should be diminished, and when it is deficient, labor should be discontinued. The light blue of the sky and the verdure of the fields are the colors to which the organ of vision is naturally adapted, and which it will endure with most ease. The flame of a good oil lamp is more regular than that of gas or candles, and is therefore to be preferred. The intermittent flickering of gas is particularly injurious, as it produces constant contractions and dilations of the pupil, and undue exercise of the whole organ. By placing a shade of light blue tissue paper over the lamp, the light is ameliorated; for artificial light contains a superabundance of the yellow an red rays, but is deficient in the violet. By allowing it to pass through the bluish medium, it approaches nearer to the light of day, and is better adapted for continuel application of the organs of vision.

The gist of the whole matter is just this. Let your eyes alone, and they may serve you all your days. Should they become out of order, apply to that very important personage, your family physician, and he will **instruct you how** to "MIND YOUR EYES."—*Scalpel.*

